# The use of Horizon graphs to visualize bilateral biomechanical time-series of multiple joints

**DOI:** 10.1016/j.mex.2021.101361

**Published:** 2021-04-24

**Authors:** Liria Akie Okai-Nobrega, Thiago Ribeiro Teles Santos, Milton Hirokazu Shimabukuro, Renan Alves Resende, Thales Rezende de Souza, Sérgio Teixeira da Fonseca

**Affiliations:** a Graduate Program in Rehabilitation Science, Department of Physical Therapy, Universidade Federal de Minas Gerais, Belo Horizonte, MG, Brazil; b Department of Mathematics and Computer Science, School of Technology and Applied Sciences, Universidade Estadual Paulista, Presidente Prudente, SP, Brazil

**Keywords:** Movement analysis, Clinical, Running, Data visualization

## Abstract

Movement analysis provides a vast amount of data, which, frequently, are not used in the clinical decision-making process. For example, traditional gait data visualization is based on a time-based display of joint angles, but part of the information is lost when these time-series are averaged across different gait strides. Horizon graph is a data display method that increases the density of time-series data by horizontally dividing and layering multiple filled line graphs. This higher data density increases the amount of information displayed in the same graph and, consequently, enables visual data comparisons between multiple time series. Horizon graph of kinematic data allows displaying several cycles of different joints and their respective continuous symmetry ratio between sides. The aim of this work is to introduce the Horizon graph as a method to analyze kinematic gait data and help to characterize its symmetry. Examples of Horizon graph application to running is offered. Horizon graph may prove to be a useful clinical tool to visualize kinematic time-series and facilitate their clinical interpretation.

• Continuous gait time series is a powerful tool for clinical analysis.
• Horizon graph, higher data density graph, increases the information displayed.
• Horizon graph is a clinical tool to visualize kinematic curves.

## Graphical abstract

**Figure:**
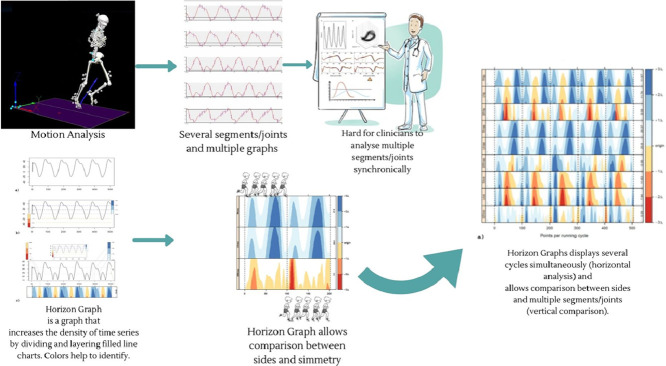

Specifications Table

**Table:** 

Subject Area:	Medicine and Dentistry
More specific subject area:	*Physical Therapy, Movement Analysis;*
Method name:	*Horizon Graph*
Name and reference of original method:	*Horizon graph* (HG) is a graph that increases the density of time series by dividing and layering filled line charts [1,^11^,12]. This higher data density increases the amount of information displayed at the same graph and allows to compare data across multiple charts. This method to visually analyze data has been used in economics, political science, and public policymaking [1]. HG could be applied to kinematic data in biomechanics to display several bilateral cycles of different joints and their respective continuous symmetry ratio.
Resource availability:	*If applicable, include links to resources necessary to reproduce the method (*e.g. *data, software, hardware, reagent)*

**Method details**

## Background - human movement analysis

Movement analysis is an essential part of biomechanical research [3,^8^,^15^,18]. However, the use of movement analysis in clinical practice is still challenging. The biomechanical analysis produces a vast amount of data, making it difficult to obtain clinically relevant information [3,^4^,14]. Also, to interpret these data, discrete variables are usually extracted from continuous time-series, which causes vital information to be lost [9,21]. Methods that allow direct visualization of the continuous movement pattern, and its symmetry, may facilitate the identification of altered movement patterns or the documentation of patient's evolution [7,^16^,17]. This work aims to present the HG as a method to analyze lower limbs' kinematic gait data and characterize its symmetry. HG may prove to be a powerful clinical tool to visualize kinematic time-series that could help clinicians interpret patient data.

## Horizon graph as a visualization tool

**Fig. 1:**
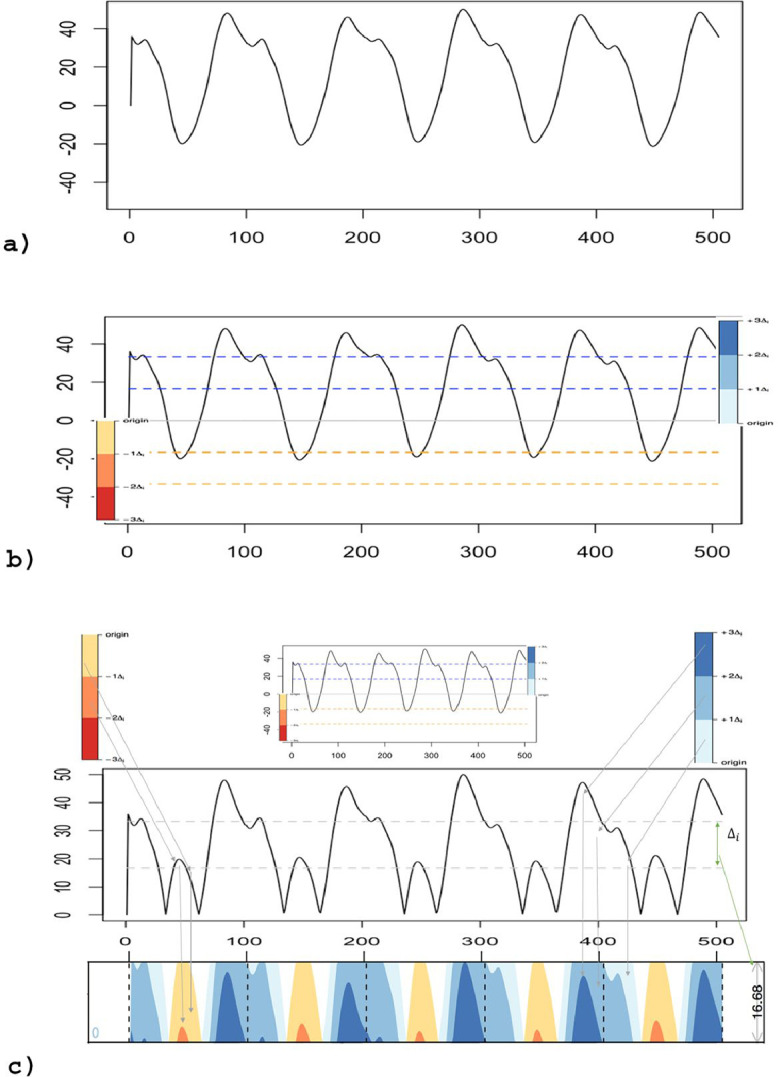
Illustration of how to plot a Horizon Graph. (a) Typical line chart of hip sagittal plane kinematic data during running. (b) Data is set to baseline (0) and divided into three-line charts with different saturation colors. (c) The data are reflected around the baseline and then layered (bottom of the figure). Δ_i_ indicates the amplitude of the line charts. The saturation corresponds to the time-series peaks, in which darker colors represent the level (magnitude) (For interpretation of the references to color in this figure, the reader is referred to the web version of this article.).

HG is constructed as showed in Fig. 1. A line chart is segmented (Fig. 1a) along the vertical axis into uniformly sized and non-overlapping bands (Fig. 1b). The bands are then layered on top of each other, and negative values are reflected around the baseline (Fig. 1c). Colors indicate positive (blue) or negative (red/yellow) values, and their intensity indicates band level (magnitude). HG reduces the height of a line chart with positive and negative values by a factor of three, which is reflected by bands. This increase in data density enables displaying more information in a fixed area and allows visual comparison of data across multiple line charts [11,12]. In HG, data values are represented not only by their vertical height (amplitude) but also by their color saturation and hue [19].

Kinematic gait time-series are highly, but not precisely, periodic and symmetrical [9,^13^,14]. They exhibit both temporal and spatial variations within and across strides [9]. These naturally occurring variations could be more effectively noted if the similarity between curves (sides) are displayed [5]. To make more evident the presence of these side variations, a symmetry ratio (SR) was implemented as such:

$$(SR=\left(R-L)/ROM_{pp}\right)$$

where:

*R* = right joint angle

*L* = left joint angle

*RoM_pp_* = Peak to peak range of motion

The use of *RoM_pp_* allows detecting side-to-side asymmetries considering magnitude related to a specific joint. The advantage is to avoid artificial inflation [5], which refers to irrelevant differences between sides that are overestimated when the denominator of a similarity index is too small or zero.

**Fig. 2:**
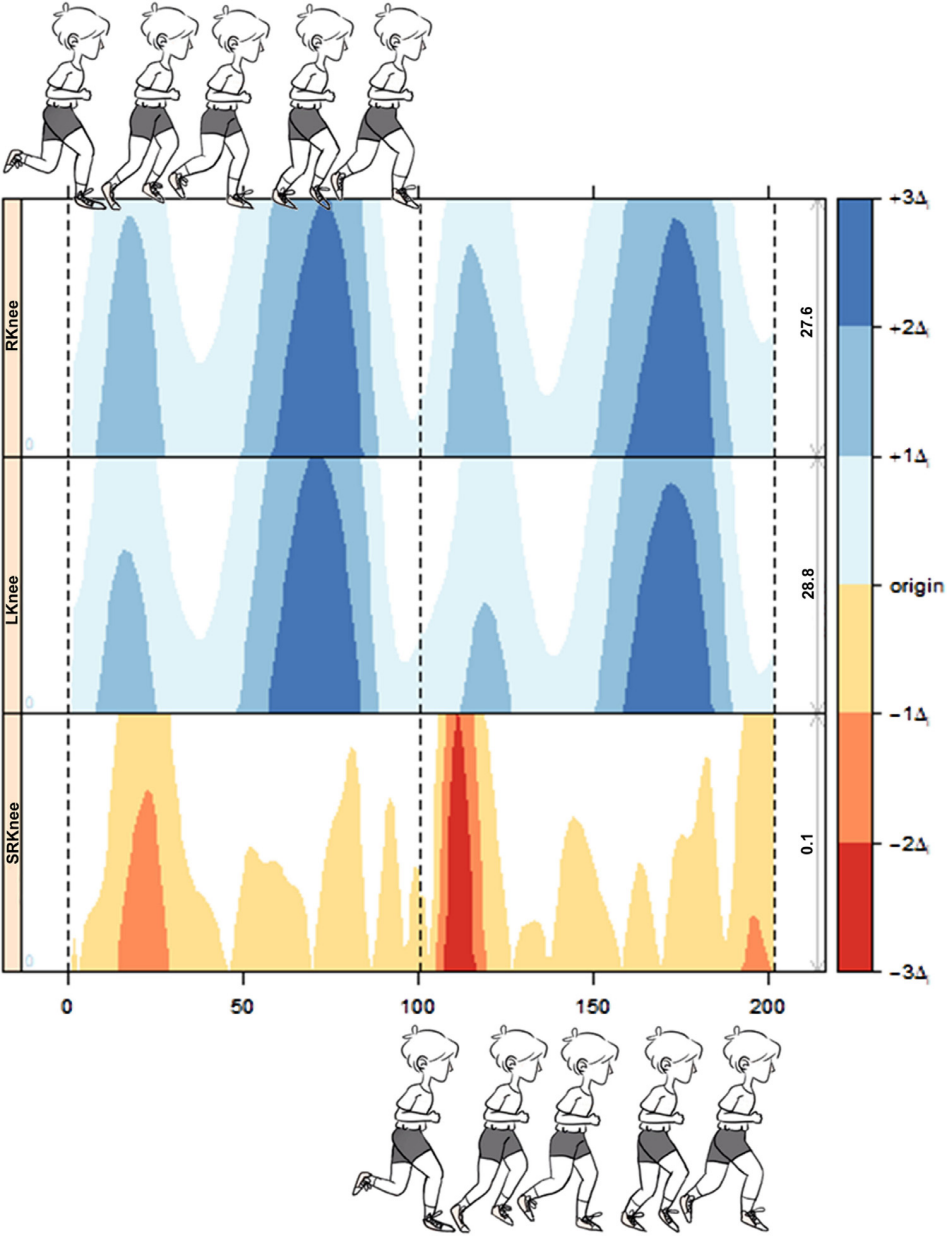
Example of horizon graph with two running time-series (right knee, left knee, and knee symmetry ratio). Each running cycle is normalized to 101 data points. The horizontal axis represents two concomitants running cycles. The left vertical axis represents the joints (right and left knees) behavior and the time-series continuous symmetry ratio. The right vertical axis represents the amplitude of the time-series band level (magnitude). The blue color represents knee flexion (i.e., positive values), and the red color represents knee extension (i.e., negative values). For knee symmetry ratio, red/yellow represents left side dominance. Color intensity indicates the peak amplitude. The vertical dotted lines separate the running cycles. Running phases are pictured: stance phase (initial contact to impulse) and swing phase (For interpretation of the references to color in this figure legend, the reader is referred to the web version of this article.).

HG displays several cycles simultaneously (horizontal comparison) and allows comparison between sides (vertical comparison). To illustrate, Fig. 2 shows HG of two cycles of sagittal kinematic data of the knee joint: right, left, and symmetry of the corresponding running cycles. The colored bar (displayed on the right side) represents the intensity of the three layers of the data. The SR time-series (3rd layer) indicates the position and intensity of side differences (blue indicates the right side having a higher angle magnitude and red indicates the left side having a higher angle magnitude). The color intensity indicates the asymmetry level. Each running cycle (stance and swing phases) is normalized in 101 data points. Therefore, the horizontal axis represents two cycles or 202 data points. The baseline was set to zero degrees [12] for all time-series. This process considered the normalization of joint angles obtained during quiet stance. The HG was generated based on an algorithm implemented in R and available as Supplementary Material.

## Clinical application of Horizon graph

To demonstrate the application of HG to biomechanical time-series, we analyzed the running data of two participants. One runner (age: 28 years old, height: 1.61 m; mass: 56.1 kg) was asymptomatic and without a recent history of injury (non-injured runner), and the other (age: 29 years old, height: 1.63 m; mass: 71.9 kg) was also asymptomatic, but with a history of a right shank stress fracture in the last year (injured runner). The subjects signed an informed consent form approved by the university's ethical committee (ETIC 0526.0.203.000-10).

Data at self-selected running speed over a treadmill were collected using three position scanner units at 100 Hz (CODA system Charnwood Dynamics, Leicestershire, England). The angular displacement of the hip, knee, and ankle joints in the sagittal plane were calculated using Visual 3D (C-Motion Inc, Germantown, MD, USA) based on a typical rigid-body model [2,6]. Positive values indicate hip and knee flexion and ankle dorsiflexion, and negative values indicate hip and knee extension and ankle plantar flexion. To analyze the HG of both subjects, we compared three specific features [19]: MAX (time series peaks for each joint), SIMI (describes how similar are the right and left time series of each joint, as measured by the SR), and DISC (discriminate visual relation of the peaks among all three joints).

**Fig. 3:**
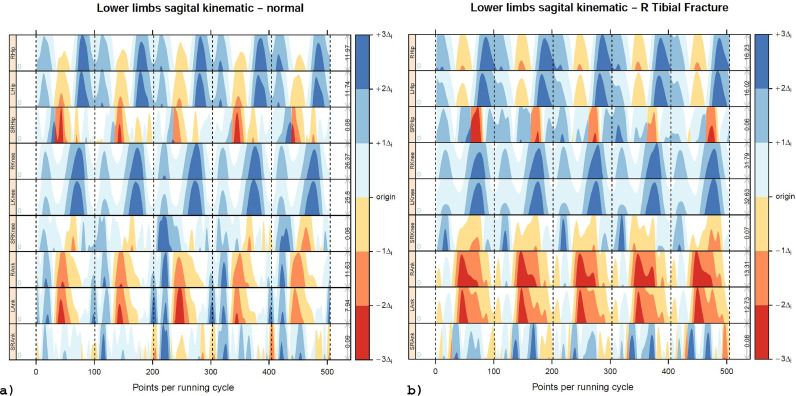
Horizon Graph of representative six time-series of lower limb running kinematic data and corresponding symmetry ratio of two subjects (one asymptomatic subject (a) and one asymptomatic subject, but with a history of a shank stress fracture in the last year (b)). Each running cycle has 101 data points. The horizontal axis represents five running cycles. The three upper graphs represent kinematic data of the right hip, left hip, and corresponding hip symmetry ratio. The three middle graphs represent kinematic data of the right knee, left knee, and corresponding symmetry ratio. The three lower graphs represent kinematic data of the right ankle, left ankle, and corresponding symmetry ratio. The blue color indicates flexion/dorsiflexion, and the red/yellowish color indicates extension/plantar flexion. For each joint symmetry ratio, blue represents right side dominance, and red represents left side dominance. The vertical dotted lines separate the running cycles (For interpretation of the references to color in this figure legend, the reader is referred to the web version of this article.).

Fig. 3 shows data from both runners. Fig. 3a shows the HG for the non-injured runner. *Hip Joint: MAX* - flexion peak at the swing phase and extension peak close to push*-*off*. SIMI* - hip SR indicated persistent asymmetry peaks at push-off, with the left hip demonstrating greater hip extension (red) than the right one. A persistent asymmetry peak is also observed in the middle of the swing phase. At this phase, the left hip had the greatest flexion angle. *Knee Joint: MAX -* peak flexion was observed at the swing phase. *SIMI:* at the first peak of knee flexion, the right knee had the greatest joint angle. At the second knee flexion peak, the left side had the greatest angle. This behavior suggests that the right side is absorbing more impact at the stance phase, and the left side is flexing more during the swing phase. *Ankle Joint: MAX -* dorsiflexion peak was observed in the middle of the stance phase, and plantarflexion peak at push-off. *SIMI:* The SR indicated the presence of asymmetry during the stance phase, with the right side having the greatest peak of dorsiflexion. When observing the relationship among all joints (*DISC*), the presence of flexion peaks of all three joints during the Stance Phase was observed. Flexion also occurred at the swing phase according to the following temporal sequence: knee, hip, and ankle. The Hip and Ankle joints had extension peaks at push-off, as expected in running cycle dynamics.

Fig 3b shows the HG of the injured runner. *Hip Joint: MAX* – was similar to the non-injured runner. *SIMI* - hip SR indicated persistent asymmetry peaks at the stance phase, with the right hip having a greater magnitude of hip flexion (blue) than the left hip. Another persistent asymmetry peak was observed at the swing phase. The left side had a greater flexion angle. *Knee Joint: MAX -* was similar to the non-injured runner. *SIMI:* demonstrated a pattern similar to the non-injured runner, except for greater asymmetry between sides. *Ankle Joint: MAX -* plantarflexion peak was observed at push-off, and dorsiflexion peak happened in the middle of the stance phase*. SIMI -* the SR indicated asymmetry during the stance and swing phases, with the right side having larger plantarflexion peaks. *DISC -* there were flexion peaks at hip and knee joints in the stance and swing phases. Similar to the non-injured runner, the hip and ankle joints had extension peaks at the push-off. However, the ankle demonstrated prolonged plantarflexion during the swing phase.

## Review capacity and clinical applications

HG is a method for visualization of quantitative and qualitative information based on synchronized multiple time series. This method allows parallel and simultaneous analyses of bilateral kinematic time-series. Simultaneous inspection of several cycles and different joints can help to detect the occurrence of atypical and asymmetrical gait patterns. A critical aspect of proper analysis of the subject's kinematic data is to characterize the locomotion pattern without constraining the mean behavior analysis. Human locomotion's cycle-to-cycle dynamics contain rich information regarding an individual [14,21]. This information is lost when the time-series is reduced and analyzed by averaging the data across strides [9,^10^,^14^,21]. Thus, the HG method's advantages are: (i) continuous visualization of movement patterns, (ii) simultaneous visualization of bilateral kinematic data of different joints, and (iii) easy identification of lower limb asymmetries. The visualization of multiple gait cycles allows identifying different gait patterns, showing regular asymmetries that can be used to guide specific clinical assessments. Thus, the HG can help guide clinical research and support the interpretation of the clinical data [11].

It was clear from the two participants' cycle-to-cycle analysis that the gait pattern was regular, but not identical, even when considering the same subject. The presence of asymmetry peaks in all joints in both subjects shows side differences that should be considered during clinical assessment. In case of differences in SR, peaks are persistent during several cycles, and the asymmetrical pattern becomes evident. In the example, the HG of the injured runner showed an atypical behavior, with ankle dorsiflexion happening only in the middle of the stance phase and plantarflexion during all swing phases. Considering MAX and SIMI features, the injured runner's hip and knee joints had movement patterns similar to the non-injured runner. Considering all three joints together, the injured runner did not show the triple flexion pattern at the stance phase and at the end of the swing phase, as the non-injured runner. This atypical motion may indicate altered mobility due to inadequate rehabilitation or modified motor pattern adopted after injury. Although the HG did not provide quantitative information for analyzing running data, data visualization allowed fast and straightforward identification of significant kinematic differences between the subjects. The possibility of multiple joints comparison allows the clinician to observe lost features when considering just one joint at once or when only discrete data are compared.

Despite the benefits offered by HG, some limitations must be indicated. The analysis is limited to a few cycles (e.g., five cycles) since plotting several cycles could be challenging and difficult to visualize. Also, processing and full implementation of the method may not be straightforward [19]. Fortunately, online tools and programming codes for implementing the method are freely available to users [20]. Furthermore, we added the algorithm implemented in R as Supplementary Material to help researchers interested in the method.

The use of HG to analyze bilateral biomechanical time-series of multiple joints can provide useful and reliable information about subjects’ behavior during walking and running. The HG plots may be used as the first step to select discrete variables that could quantify the finding that the researcher or clinician judge as relevant to further analysis. Together with the simultaneous joint kinematics' visual information, the presentation of a continous symmetry ratio may provide useful information in a clinical setting, such as evaluation in the beginning and during an intervention, and pre-season sports evaluation. Despite the fast visualization of meaningful features of kinematic data, full acceptance of the method will depend on the clinicians’ impressions regarding its applicability to support clinical decision-making. Therefore, the proposed method applies a procedure new to biomechanics that has the potential to optimize simultaneous graphical visualization of bilateral kinematic waveforms of multiple joints.

## Declaration of Competing Interest

The authors declare that they have no known competing financial interests or personal relationships that could have appeared to influence the work reported in this paper.

The authors declare the following financial interests/personal relationships which may be considered as potential competing interests:

## Appendix Supplementary materials

Image, application 1
